# Diagnostic Yield and Safety of Radial Probe Endobronchial Ultrasound-Guided Transbronchial Lung Cryobiopsy with a Guide Sheath in Pulmonary Lesions < 3 cm

**DOI:** 10.3390/diagnostics16121912

**Published:** 2026-06-19

**Authors:** Taehun Kim, Yujin Lee, Jung Hee Hong, Seong Hwan Youn, Hyun Jung Kim, Jae Seok Park, Sun Hyo Park

**Affiliations:** 1 Division of Pulmonary Medicine, Department of Internal Medicine, Keimyung University Dongsan Hospital, Keimyung University School of Medicine, Daegu 42601, Republic of Korea; spmemory88@gmail.com (S.H.Y.); khj82827@gmail.com (H.J.K.); jspark1025@dsmc.or.kr (J.S.P.); ibagu70@gmail.com (S.H.P.); 2 Department of Internal Medicine, Keimyung University Dongsan Hospital, Keimyung University School of Medicine, Daegu 42601, Republic of Korea; jfmamjj89@gmail.com; 3 Department of Radiology, Keimyung University Dongsan Hospital, Keimyung University School of Medicine, Daegu 42601, Republic of Korea; keke4@naver.com

**Keywords:** radial probe endobronchial ultrasound, transbronchial lung cryobiopsy, guide sheath, target-fixing technique

## Abstract

**Background/Objectives**: Accurate tissue diagnosis of small pulmonary nodules remains technically challenging with conventional bronchoscopic techniques. Radial probe endobronchial ultrasound-guided transbronchial lung cryobiopsy (RP-EBUS–guided TBLC) with a guide sheath (GS) may improve diagnostic yield; however, target instability during cryobiopsy remains a limitation. We aimed to evaluate the diagnostic yield of RP-EBUS-guided TBLC with a GS for pulmonary nodules < 3 cm that were suspected of malignancy. **Methods**: This retrospective observational study included patients who underwent RP-EBUS-guided TBLC with a GS for lung lesions suspected of malignancy on computed tomography between 1 February 2024 and 31 December 2025 in South Korea. After the target lesion was identified, the bronchoscope was inserted and fixed within the segment; its position was maintained while RP-EBUS was withdrawn, and lesion stability during respiration was confirmed. **Results**: A total of 99 patients were included in the final analysis. After patients with an indeterminate diagnosis were excluded, the final diagnostic yield was 83.2%. The sensitivity and specificity were 78.9% and 100.0%, respectively. Pneumothorax occurred in 6.0% (6/99) of patients. Bleeding of grade 3 or higher was observed in two patients, and a Fogarty balloon catheter was preemptively used in five patients at the operator’s discretion. In multivariable logistic regression analysis, the computed tomography bronchus sign was identified as the only significant factor associated with pathological confirmation (odds ratio, 6.090; *p* = 0.005). **Conclusions**: RP-EBUS-guided TBLC with a GS provided an acceptable diagnostic yield and safety profile, even in small pulmonary nodules < 3 cm.

## 1. Introduction

Globally, lung cancer remains the most commonly diagnosed malignancy and the leading cause of cancer-related mortality, with an estimated 2.48 million new cases and 1.8 million deaths reported in 2022 [1].

Low-dose computed tomography (LDCT) screening in individuals at high risk has become a key strategy for reducing lung cancer-related mortality [2]. In selected patients with a high suspicion of malignancy, surgical resection may be performed without prior biopsy. Among patients with lung nodules suspected of malignancy, upfront video-assisted thoracoscopic surgery has resulted in a final non-cancer diagnosis in 15–34.1% of patients [3,4,5]. A recent nationwide population-based study reported that the use of self-initiated, non-reimbursed LDCT screening during health check-ups markedly increased despite low eligibility for risk-based screening, leading to more diagnoses of localized lung cancer without a reduction in distant-stage disease and suggesting potential overdiagnosis and increased healthcare utilization [6]. Therefore, accurate pathological confirmation before treatment is initiated has become essential to guide optimal management and avoid inappropriate interventions [3]. The broader adoption of molecular profiling, such as next-generation sequencing, has increased the demand for tissue biopsies [7], further underscoring the importance of obtaining high-quality samples from peripheral pulmonary lesions (PPLs).

For the diagnosis of PPLs, non-surgical biopsy techniques, including CT-guided percutaneous lung biopsy and bronchoscopic biopsy, have been developed. The diagnostic yield of CT-guided percutaneous lung biopsy is approximately 83.5%, exceeding that of radial probe endobronchial ultrasound (RP-EBUS), particularly for lesions 1–2 cm in size (83% vs. 50%) [8]. Despite its diagnostic utility, CT-guided biopsy is associated with procedure-related complications, with pneumothorax occurring in up to 61% of patients and hemorrhage reported in 5–16.9% of patients [9]. Although achieving a high diagnostic yield remains a challenge, particularly for smaller nodules, bronchoscopic methods for PPL biopsy, including RP-EBUS, guide sheath (GS), and electromagnetic navigation bronchoscopy (ENB), have been developed [10]. The overall diagnostic yields of CT-guided lung biopsy and bronchoscopic biopsy have been reported to be 88.9% and 73.9%, respectively. By comparison, robot-assisted bronchoscopy (RAB) has recently shown a diagnostic yield of approximately 84.8% [11]. The overall diagnostic yield of RP-EBUS-guided transbronchial lung biopsy (TBLB) without fluoroscopy was 70% (95% CI, 67–74%). For lesions < 3 cm, the diagnostic yield was 62% (56–67%). After the introduction of cryobiopsy, meta-analyses showed diagnostic yields of 77% (95% CI, 71–84%) for cryobiopsy and 72% (95% CI, 60–83%) for forceps biopsy [12,13]. However, studies reporting the evaluation of lesions < 3 cm remain limited. In this study, we performed RP-EBUS-guided transbronchial lung cryobiopsy (TBLC) without fluoroscopy and applied a target-fixing technique [14] to overcome the limitations of the semi-real-time procedure. We evaluated the diagnostic performance of this approach for PPLs measuring < 3 cm, as well as factors potentially associated with diagnostic outcomes and cryobiopsy results.

## 2. Materials and Methods

### 2.1. Study Design and Patient Selection

**Figure 1:**
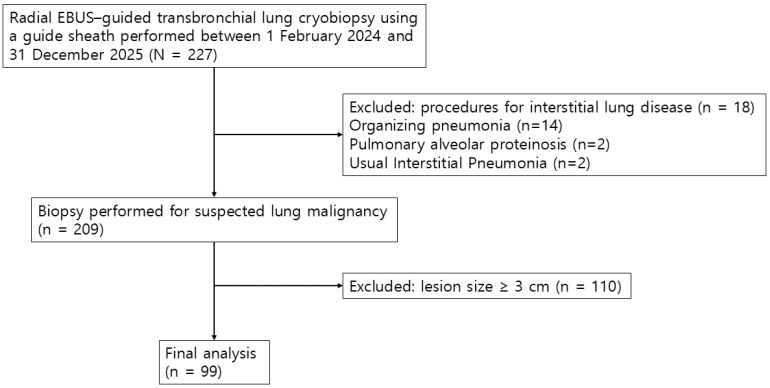
Flow diagram illustrating the patient selection process. EBUS, endobronchial ultrasound.

In this retrospective observational study, we analyzed clinical data from 227 patients who underwent RP-EBUS-guided sheath-assisted cryobiopsy without fluoroscopy at a tertiary medical center in South Korea between 1 February 2024 and 31 December 2025. Patients with lesions suspected of malignancy were included. A total of 110 patients with lesions ≥ 3 cm and 18 patients who underwent biopsy for interstitial lung disease were excluded. The final analysis included 99 patients (Figure 1). PPLs were defined as pulmonary lesions located within the lung parenchyma that could be detected on CT but were not directly visible during bronchoscopy.

### 2.2. Target Fixing Method (Dohyun’s Method)

All procedures were performed using Dohyun’s method, also referred to as the “target-fixing technique,” as previously described [14]. Briefly, after the target lesion was identified using RP-EBUS, the bronchoscope was advanced to stabilize its position, and the RP-EBUS probe was carefully withdrawn while maintaining the bronchoscope position established during visualization of the target throughout the respiratory cycle. Tissue sampling was then performed while maintaining the most stable sampling position possible.

### 2.3. Procedure

All bronchoscopic procedures were performed by a pulmonologist (THK). Patients received intravenous midazolam (2–5 mg) and fentanyl (25–50 μg) for moderate-to-deep conscious sedation at the start of the procedure. No mechanical ventilation was used during the intervention. Following completion of bronchoscopy, flumazenil (0.2 mg intravenously) was routinely administered to reverse the sedative effects of midazolam. All procedures were performed under endotracheal intubation using a 7.0 mm endotracheal tube, with oxygen supplied at 2–6 L/min through the endotracheal tube. Local anesthesia was achieved by instilling lidocaine hydrochloride solution (1%, 200 mg/20 mL) onto the vocal cords, carina, and both main bronchi via the bronchoscope (BF-P290; 2.0 mm working channel; Olympus, Tokyo, Japan).

The target PPL was accessed using a 1.4 mm RP-EBUS (UM-S20-17S; Olympus) in combination with a 1.95 mm guide sheath (K-201 or K-402; Olympus). Once the lesion was visualized with RP-EBUS, the target was considered successfully localized. After target stabilization using Dohyun’s method, the RP-EBUS probe was withdrawn, and a cryobiopsy probe (flexible cryoprobe; outer diameter 1.1 mm; length 1.15 m; Erbe Elektromedizin GmbH, Tübingen, Germany) was advanced through the GS. Tissue samples were obtained by freezing for 4–8 s, after which the cryoprobe and bronchoscope were removed together to retrieve the specimen.

Post-biopsy bleeding was immediately evaluated bronchoscopically through the endotracheal tube, and epinephrine was administered as needed to achieve hemostasis. If preprocedural CT findings suggested a high risk of bleeding, a hemostatic balloon catheter (B5-2C; Olympus) was positioned in advance within the target segment and inflated immediately after sampling. Additional cryobiopsy attempts were performed at the operator’s discretion. In cases of severe bleeding that was not controlled with conservative measures, the hemostatic balloon catheter was used for tamponade.

### 2.4. Data Collection

Data were retrospectively extracted from electronic medical records. The following variables were extracted and analyzed: (1) patient demographic and clinical characteristics, including sex, age, height, weight, comorbidities, and smoking history; (2) pulmonary function parameters, including forced expiratory volume in 1 s (FEV1), forced vital capacity (FVC), and the FEV1/FVC ratio; (3) chest CT findings, including lesion size, lesion type (solid or sub-solid), pleural contact, lobar location, and axial distribution; (4) RP-EBUS findings, categorized by probe position as “within” vs. “adjacent to” and by echogenic pattern as “dense sign” vs. “blizzard sign” [15]; (5) procedural details, including procedure duration, number of biopsies, use of epinephrine for hemostasis, and adverse events such as pneumothorax; and (6) pathology results, including the biopsy diagnosis and confirmation of the final diagnosis using follow-up CT or surgical biopsy.

Computed tomography bronchus sign (CT-BS) was recorded as either present (airway adjacent to or aligned with the target) or absent. Axial distribution was divided into inner, middle, and outer thirds, whereas vertical distribution was classified as the upper lung region (right and left upper lobes and right middle lobe) or the lower lung region (right and left lower lobes). Lesion size was measured as the longest diameter on CT. Lesions were categorized as solid, sub-solid, or ground-glass opacity; none of the included lesions were ground-glass opacities. CT imaging findings were reviewed and classified by consensus between a pulmonologist (THK) and a thoracic radiologist (JHH). The number of biopsies was defined as the total number of tissue samples obtained per patient. Bleeding was graded on a 4-point scale based on bronchoscopic findings and the interventions required, ranging from grade 1 (minimal bleeding requiring brief suction) to grade 4 (severe bleeding requiring advanced interventions such as prolonged intubation, transfusion, or bronchial artery embolization) [15].

### 2.5. Statistical Analysis

Categorical variables are summarized as frequencies and percentages, whereas continuous variables are summarized as means ± standard deviations. Diagnostic yield was defined as the proportion of patients with true-positive or true-negative (TN) results in the total population [15]. For cases without pathological confirmation, TNs were defined as lesions that showed improvement on follow-up CT after ≥6 months, were confirmed as tuberculosis with resolution following anti-tuberculosis therapy, or were surgically proven granulomas. Patients with initially negative results but insufficient follow-up or those who declined further evaluation were considered to have an indeterminate diagnosis. These cases were included in sensitivity analyses, with the assumption that all were either TNs or false negatives (FNs). Lower- and upper-bound estimates of diagnostic yield, sensitivity, specificity, positive predictive value, and negative predictive value were calculated.

Associations between nominal variables were evaluated using the chi-square test. Multivariable logistic regression analysis was conducted to identify factors associated with pathological confirmation. Variables with *p* < 0.2 in univariate analyses were included in the multivariable model, which was built using backward stepwise selection based on the log-likelihood ratio. A *p*-value < 0.05 was considered statistically significant. All analyses were performed using IBM SPSS Statistics, version 25 (IBM Corp., Armonk, NY, USA), and R version 4.1.1.

### 2.6. Ethics Statement

The study was reviewed and approved by the Institutional Review Board of Keimyung University College of Medicine (approval no. 2025-12-086). The study was conducted in accordance with the principles of the Declaration of Helsinki (2013 revision). The requirement to obtain informed consent was waived because of the retrospective design of the study.

## 3. Results

### 3.1. Baseline, Lesion, and Procedural Characteristics

**Figure 2:**
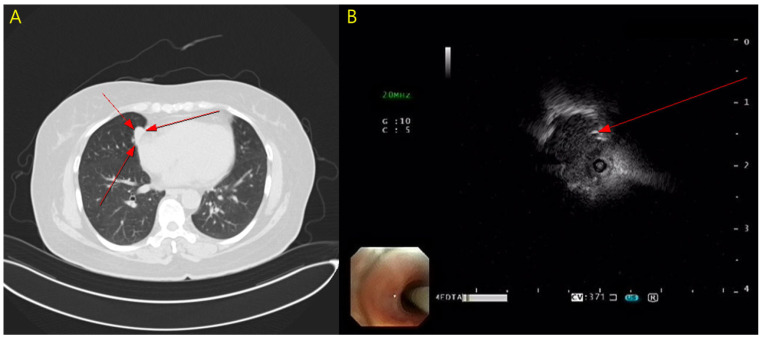
Representative case of successful RP-EBUS localization in a pulmonary nodule without CT-BS: (**A**) Chest CT demonstrating a pulmonary nodule (arrow) without an identifiable CT bronchus sign. (**B**) RP-EBUS image showing a dense sign with an adjacent orientation relative to the target lesion (arrow). RP-EBUS, radial probe endobronchial ultrasound-guided; CT-BS, computed tomography bronchus sign.

Data from 99 patients who underwent RP-EBUS-GS TBLC were evaluated (Figure 1). Table 1 summarizes the patient characteristics. The mean age was 68.1 ± 10.1 years; 52 (52.5%) patients were men, and 46 (46.5%) were classified as ever-smokers. The most common location of the target lesion was the left upper lung region (32.3%), followed by the right upper lung region (28.3%). The mean lesion size on CT was 2.50 ± 1.20 cm, and 27 (22.5%) lesions were classified as sub-solid nodules. With regard to axial distribution, lesions were located in the inner third (two lesions, 2.0%), middle third (54 lesions, 54.5%), and outer third (43 lesions, 43.4%). CT-BS was present in 81 (81.8%) patients (Table 1). RP-EBUS findings were classified as “within” in 49 (49.5%) patients, and a dense sign was observed in 64 (64.6%) patients. The mean number of TBLC specimens obtained was 3.4 ± 1.2, and the procedure time, measured from sedative administration to antidote administration, was 31.7 ± 11.5 min. Even in nodules without a CT-BS (Figure 2A), some lesions could be successfully visualized using RP-EBUS by meticulously advancing the guide sheath and radial probe through each subsegmental bronchus in a stepwise manner. This systematic one-by-one exploration enabled localization of the target lesion and acquisition of characteristic RP-EBUS images (Figure 2B).

### 3.2. Diagnostic Outcomes and Complications

**Figure 3:**
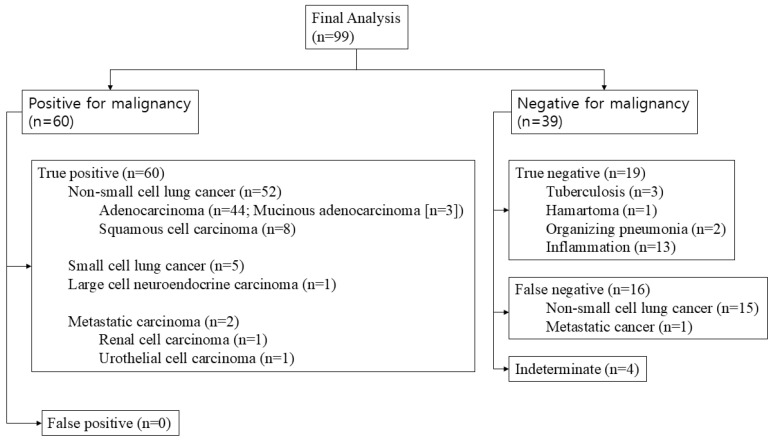
Diagnostic outcomes of the study patients.

The final diagnostic results, including diagnoses not confirmed by RP-EBUS-guided TBLC, are presented in Figure 3. Malignancy was confirmed by RP-EBUS-guided TBLC in 60 (60.6%) of 99 patients. Among patients with RP-EBUS-guided TBLC-confirmed malignancy, non-small cell lung cancer was the most common diagnosis (52 patients), with adenocarcinoma as the predominant subtype (44 patients). Of the patients without malignancy confirmed by RP-EBUS-guided TBLC, 16 were diagnosed with malignancy by CT-guided percutaneous lung biopsy or surgical lung biopsy. Nineteen patients were classified as having TN results; 13 were confirmed to have inflammation, which resolved within 6 months on follow-up CT; one had a hamartoma, two were diagnosed with organizing pneumonia, and three were diagnosed with tuberculosis, which improved following anti-tuberculosis treatment. Four patients were not initially diagnosed with malignancy and were not further evaluated owing to loss to follow-up or refusal to undergo further invasive workup.

The final diagnostic yield of RP-EBUS-guided TBLC for lesions < 3 cm was 83.2%, excluding patients with an indeterminate diagnosis (Table 2). When an indeterminate diagnosis was considered either an FN or a TN result, the diagnostic yield ranged from 79.8% to 83.8%. The sensitivity and specificity were 78.9% and 100.0%, respectively (Table 2).

Pneumothorax occurred in 6.0% (6/99) of patients, and three patients required chest tube placement. A Fogarty balloon catheter was preemptively placed in five patients at the operator’s discretion. Grade ≥ 3 bleeding occurred in five patients; however, none required intensive care unit care, embolization, or selective intubation, and no bleeding-related deaths occurred (Table 1).

### 3.3. Factors Associated with Higher Diagnostic Yield

Comparison between diagnostic and non-diagnostic cases revealed no significant differences in lesion size, distribution, or nodule characteristics, except for the presence of a CT-BS (Supplementary Table S1).

Univariate and multivariate analyses of factors associated with diagnostic confirmation are shown in Table 3. In the univariate analysis, only the presence of CT-BS was associated with a higher diagnostic yield. In the multivariate analysis, after adjustment, CT-BS (odds ratio, 6.090; *p* = 0.005) was identified as a significant factor associated with diagnostic confirmation (Table 3).

## 4. Discussion

We retrospectively evaluated the diagnostic yield of RP-EBUS-guided TBLC using a target-fixing method for target lesions < 3 cm. The diagnostic yield of RP-EBUS-GS TBLC was 83.2%, indicating that RP-EBUS-guided TBLC may be a reliable diagnostic method for small peripheral lung lesions.

With advances in bronchoscopic biopsy techniques, recent studies have reported the comparison of diagnostic yields across different modalities. A previous meta-analysis reported a diagnostic yield of 70% for RP-EBUS-guided forceps biopsy [16]. For ENB, the reported diagnostic yield was 72.7% [11]. In lesions ≤ 15 mm, T. Ito et al. reported that endobronchial ultrasonography-guided transbronchial biopsy achieved a diagnostic yield of 77.5% (62/80) [17]. However, in a meta-analysis, the diagnostic yield for lesions < 3 cm was 62% (56–67%) [11]. After the introduction of cryobiopsy, meta-analyses showed diagnostic yields of 77% (95% CI, 71–84%) for cryobiopsy and 72% (95% CI, 60–83%) for forceps biopsy [12,13]. One study reported higher diagnostic performance with ENB than with RP-EBUS-guided TBLB (83.8% vs. 70.5%, *p* = 0.021) [18]. However, a meta-analysis comparing ENB with RP-EBUS alone showed no significant difference in diagnostic performance (relative risk 1.12, 95% CI, 0.95–1.33) [11]. A recent meta-analysis revealed that the addition of RP-EBUS did not significantly improve diagnostic yield when combined with virtual bronchoscopy (*p* = 0.36) or ENB (*p* = 0.70), whereas the addition of RAB resulted in a significant improvement in diagnostic yield (85.1% vs. 76.9%, *p* = 0.02). These findings suggest that the role of RP-EBUS may extend beyond simple lesion localization. Although navigation platforms can facilitate access to the target lesion, RP-EBUS may help maintain accurate targeting during tissue acquisition by reducing the gap between lesion confirmation and biopsy. This concept is consistent with our target-fixation approach and highlights the importance of maintaining stable target engagement throughout the biopsy procedure to optimize diagnostic performance [11].

Recently, cryobiopsy has been increasingly applied in lung biopsy. It has been widely used for interstitial lung disease diagnosis [19] and has also been applied to lung cancer diagnosis, with a reported diagnostic yield of 77% (95% CI 71–84%) [12,20]. Cryobiopsy may also provide better diagnostic performance than forceps biopsy (forceps biopsy vs. cryobiopsy; 65.3% [130/199] vs. 84.4% [168/199]) [21].

Regarding lesion size, studies have reported diagnostic yields of 74–82% for lesions < 3 cm [21,22] and 82.5% for lesions ≤ 2 cm [14]. Furthermore, RAB has shown a diagnostic yield of 84.8% (95% CI 81.1–87.8%), and a more recent study reported a diagnostic yield of 89.0% for lesions ≤ 2 cm (138/155; 95% CI, 83.0–93.5%) [23]. In that cohort, cryobiopsy was performed in 88 patients (56.8%). These findings suggest that, beyond accurate target localization with RAB, optimization of the tissue acquisition technique may be a critical determinant of diagnostic yield [22]. Recent randomized controlled trials, although including a mixed population of patients undergoing biopsy for pulmonary nodules/masses and interstitial lung disease (192/490 and 298/490 patients, respectively), have also demonstrated superior diagnostic performance of 1.1 mm cryoprobe-guided cryobiopsy over forceps biopsy for pulmonary nodules or masses, with diagnostic yields of 83.2% and 70.1%, respectively (*p* = 0.04) [24].

In our study, 18 patients did not have CT-BS but had lesions that could be visualized by RP-EBUS (Figure 2A,B). In the multivariable analysis, when the target lesion was successfully accessed and cryobiopsy was performed using the target-fixing technique, conventional factors known to affect diagnostic yield, such as lesion size, were not significant, and only CT-BS remained a relevant factor. This may be attributable to the technical advantage of cryobiopsy, which enables the acquisition of larger and more diagnostically adequate tissue samples than forceps biopsy, particularly when the probe is precisely positioned within the target lesion.

Our study has some limitations. First, this was a retrospective single-center study. Therefore, the results require further validation in larger studies. Second, the diagnostic yield may have been overestimated because the procedure was performed in patients selected on the basis of CT findings that suggested suitability for bronchoscopy. Third, the learning curve associated with TBLC and the effects of fluoroscopy have not been fully elucidated; therefore, evidence regarding the routine initial use of this procedure and its clinical utility remains limited. Fourth, because cryobiopsy and the target-fixation strategy were applied simultaneously, the relative contribution of each component to the observed diagnostic yield could not be independently assessed. Therefore, whether the improved diagnostic performance was primarily driven by the superior tissue acquisition capability of the 1.1 mm cryoprobe, the reduction in targeting error achieved through RP-EBUS-guided target fixation, or the synergistic effect of both approaches remains unclear. Prospective studies aimed at directly comparing these strategies are warranted to clarify their individual contributions.

Despite these limitations, we focused on pulmonary nodules < 3 cm and evaluated the diagnostic performance and safety of RP-EBUS-guided TBLC performed without fluoroscopic guidance. Additionally, Dohyun’s target-fixation method was applied to maintain stable lesion targeting throughout the procedure. These findings provide meaningful evidence supporting the feasibility of fluoroscopy-free cryobiopsy for diagnosing small pulmonary nodules. Furthermore, given that the routine implementation of RAB may be constrained in certain healthcare settings because of costs, infrastructure requirements, and resource availability, this approach may represent a practical and accessible alternative for selected patients undergoing bronchoscopic biopsy under moderate sedation using conventional flexible bronchoscopy.

## 5. Conclusions

RP-EBUS-guided TBLC with a GS and target-fixing technique provided an acceptable diagnostic yield and safety profile for small pulmonary nodules < 3 cm.

## Figures and Tables

**Table 1:** Baseline characteristics of the patients.

Characteristics	Total (N = 99)
Age	68.1 (±10.1)
Male sex	52 (52.5%)
Height (cm) Weight (kg)	159.6 (±8.3) 60.6 (±10.4)
Ever-smoker	46 (46.5%)
Pulmonary function test FEV1/FVC FEV1, % pred, mean FVC, % pred, mean	0.75 (±0.10) 101.5 (±23.4) 93.1 (±17.6)
Comorbidity Hypertension Diabetes mellitus Airway disease Interstitial lung disease	29 (29.3%) 16 (16.2%) 10 (10.1%) 6 (6.1%)
Lobar location Right upper lung Right middle lung Right lower lung Left upper lung Left lower lung	28 (28.3%) 10 (10.1%) 16 (16.2%) 32 (32.3%) 13 (13.1%)
Axial distribution ^a^ Inner Middle Outer	2 (2.0%) 54 (54.5%) 43 (43.4.0%)
Lesion size on CT, cm	2.50 (±1.20)
Type of lesion, n (%) Solid Sub-solid	75 (75.8%) 24 (24.2%)
Pleural abutting Cavity	23 (23.2%) 4 (4.0%)
CT-BS Radial probe endobronchial ultrasound Within Eccentric Dense sign Blizzard sign	81 (81.8%) 49 (49.5%) 50 (50.5%) 64 (64.6%) 35 (35.4%)
Biopsy specimen Number of biopsies, n Procedure time (minute)	3.4 (±1.2) 31.7 (±11.5)
Pneumothorax	6 (6.0%)
Grade > 2 bleeding	5 (5.0%)
Bleeding control Epinephrine, n ^b^	4.2 (3.0)

Data are expressed as the mean (±standard deviation) or n (%). FEV1, forced expiratory volume in 1 s; FVC, forced vital capacity; CT, computed tomography; CT-BS, computed tomography bronchus sign. ^a^ Axial distribution was defined as the inner, middle, and outer third. ^b^ Total number of epinephrine ampules administered through the bronchoscope during the procedure.

**Table 2:** Diagnostic yield of radial probe endobronchial ultrasound-guided sheath-guided cryobiopsy.

	Excluding Indeterminate Cases (n = 95)	Low Estimate (n = 99)	High Estimate (n = 99)
Diagnostic yield [TP + TN/all patients]	79/95 (83.2%)	79/99 (79.8%)	83/99 (83.8%)
Sensitivity for malignancy [TP/TP + FN]	60/76 (78.9%)	60/80 (75.0%)	60/76 (78.9%)
Specificity for malignancy [TN/TN + FP]	19/19 (100.0%)	19/19 (100.0%)	23/23 (100.0%)
Positive predictive value [TP/TP + FP]	60/60 (100.0%)	60/60 (100.0%)	60/60 (100%)
Negative predictive value [TN/TN + FN]	19/35 (54.3%)	19/39 (48.7%)	23/39 (59.0%)

Data are expressed as n (%). TP, true positive; TN, true negative; FN, false negative; FP, false positive.

**Table 3:** Logistic regression model for pathological confirmation.

Characteristic	Univariate Analysis			Multivariate Analysis		
	OR	95% CI	*p*-Value	OR	95% CI	*p*-Value
Age Male sex Height	1.010 0.583 0.955	0.962–1.061 0.208–1.634 0.896–1.017	0.689 0.305 0.153	0.975	0.905–1.051	0.511
Ever smoking	0.956	0.351–2.604	0.930			
Type of lesion (solid)	0.308	0.105–0.905	0.832			
Lesion size on CT, cm	3.315	0.729–7.354	0.155	2.261	0.601–8.502	0.227
CT-BS	8.000	2.521–25.390	<0.001	6.090	1.737–21.355	0.005
Vertical distribution (lower)	1.562	0.467–5.224	0.469			

OR, odds ratio; CI, confidence interval.

## Data Availability

The datasets generated and/or analyzed during the current study are not publicly available because explicit patient consent for data sharing was not obtained.

## Supplementary Materials

The following supporting information can be downloaded at: https://www.mdpi.com/article/10.3390/diagnostics16121912/s1, Table S1: Comparison of baseline characteristics between diagnosed and non-diagnosed cases.

## Abbreviations

The following abbreviations are used in this manuscript:

LDCT	Low-dose computed tomography
PPL	Peripheral pulmonary lesion
RP-EBUS	Radial probe endobronchial ultrasound
GS	Guide sheath
ENB	Electromagnetic navigation bronchoscopy
TBLB	Transbronchial lung biopsy
TBLC	Transbronchial lung cryobiopsy

## References

[1] Bray F., Laversanne M., Sung H., Ferlay J., Siegel R.L., Soerjomataram I., Jemal A. (2024). Global cancer statistics 2022: GLOBOCAN estimates of incidence and mortality worldwide for 36 cancers in 185 countries. CA Cancer J. Clin..

[2] Aberle D.R., Adams A.M., Berg C.D., Black W.C., Clapp J.D., Fagerstrom R.M., Gareen I.F., Gatsonis C., Marcus P.M., National Lung Screening Trial Research Team (2011). Reduced lung-cancer mortality with low-dose computed tomographic screening. N. Engl. J. Med..

[3] Wilson D.O., Weissfeld J.L., Fuhrman C.R., Fisher S.N., Balogh P., Landreneau R.J., Luketich J.D., Siegfried J.M. (2008). The Pittsburgh Lung Screening Study (PLuSS): Outcomes within 3 years of a first computed tomography scan. Am. J. Respir. Crit. Care Med..

[4] El Alam R., Byrne S.C., Hammer M.M. (2023). Rate of benign nodule resection in a lung cancer screening program. Clin. Imaging.

[5] Armand E., Boulate D., Fourdrain A., Nguyen N.A.T., Resseguier N., Brioude G., Trousse D., Doddoli C., D’journo X.B., Thomas P.A. (2022). Benignant and malignant epidemiology among surgical resections for suspicious solitary lung cancer without preoperative tissue diagnosis. Eur. J. Cardiothorac. Surg..

[6] Kim S.Y., Silvestri G.A., Kim Y.W., Kim R.Y., Um S.W., Im Y., Hwang J.H., Choi S.H., Eom J.S., Gu K.M. (2025). Screening for lung cancer, overdiagnosis, and healthcare utilization: A nationwide population-based study. J. Thorac. Oncol..

[7] Cainap C., Balacescu O., Cainap S.S., Pop L.A. (2021). Next generation sequencing technology in lung cancer diagnosis. Biology.

[8] Ho A.T.N., Gorthi R., Lee R., Chawla M., Patolia S. (2023). Solitary lung nodule: CT-guided transthoracic biopsy vs transbronchial biopsy with endobronchial ultrasound and flexible bronchoscope, a meta-analysis of randomized controlled trials. Lung.

[9] Manhire A., Charig M., Clelland C., Gleeson F., Miller R., Moss H., Pointon K., Richardson C., Sawicka E., BTS (2003). Guidelines for radiologically guided lung biopsy. Thorax.

[10] Eberhardt R., Anantham D., Ernst A., Feller-Kopman D., Herth F. (2007). Multimodality bronchoscopic diagnosis of peripheral lung lesions: A randomized controlled trial. Am. J. Respir. Crit. Care Med..

[11] Balasubramanian P., Abia-Trujillo D., Barrios-Ruiz A., Garza-Salas A., Koratala A., Chandra N.C., Yu Lee-Mateus A., Labarca G., Fernandez-Bussy S. (2024). Diagnostic yield and safety of diagnostic techniques for pulmonary lesions: Systematic review, meta-analysis and network meta-analysis. Eur. Respir. Rev..

[12] Sryma P.B., Mittal S., Madan N.K., Tiwari P., Hadda V., Mohan A., Guleria R., Madan K. (2023). Efficacy of radial endobronchial ultrasound (R-EBUS) guided transbronchial cryobiopsy for peripheral pulmonary lesions (PPL’s): A systematic review and meta-analysis. Pulmonology.

[13] Sumi T., Oki M. (2026). Transbronchial cryobiopsy for peripheral pulmonary lesions using ultrathin bronchoscopy: A narrative review. J. Thorac. Dis..

[14] Kim T., Youn S.H., Kim M.-A., Kim H.J., Kwon Y., Park J.S., Park S.H. (2025). Outcomes of radial probe endobronchial ultrasound-guided transbronchial lung cryobiopsy using guide sheath and the target fixing technique. Transl. Lung Cancer Res..

[15] Folch E.E., Mahajan A.K., Oberg C.L., Maldonado F., Toloza E., Krimsky W.S., Oh S., Bowling M.R., Benzaquen S., Kinsey C.M. (2020). Standardized definitions of bleeding after transbronchial lung biopsy: A Delphi consensus statement from the nashville working group. Chest.

[16] Wang Memoli J.S., Nietert P.J., Silvestri G.A. (2012). Meta-analysis of guided bronchoscopy for the evaluation of the pulmonary nodule. Chest.

[17] Ito T., Matsumoto M., Kujime M., Kohnoh T., Fukushima A., Takagi T., Fukushima Y., Kasahara T. (2021). Efficacy of endobronchial ultrasound-guided transbronchial biopsy without guide sheath for small peripheral pulmonary lesions (≤15 mm): A retrospective cohort study. Clin. Respir. J..

[18] Kim Y.W., Kim H.-J., Yoon S.H., Song M.J., Kwon B.S., Lim S.Y., Lee Y.J., Park J.S., Cho Y.-J., Lee J.H. (2023). Electromagnetic navigation bronchoscopy versus radial endobronchial ultrasound for diagnosing lung cancer: A propensity score-matched analysis. Arch. Bronconeumol..

[19] Lachowicz J.A., Smallwood N.E., Prasad J.D., Patel P., Voutier C., Khor Y.H., Steinfort D.P. (2024). A systematic review of procedural and sampling techniques for cryobiopsy in interstitial lung disease. Eur. Respir. Rev..

[20] Tang Y., Tian S., Chen H., Li X., Pu X., Zhang X., Zheng Y., Li Y., Huang H., Bai C. (2024). Transbronchial lung cryobiopsy for peripheral pulmonary lesions. A narrative review. Pulmonology.

[21] Chung C., Kim Y., Lee J.E., Kang D.H., Park D. (2024). Diagnostic value of transbronchial lung cryobiopsy using an ultrathin cryoprobe and guide sheath for peripheral pulmonary lesions. J. Bronchol. Interv. Pulmonol..

[22] Seong H., Kim S.H., Mok J., Kim M.H., Lee M.K., Kim K., Eom J.S. (2025). Cryobiopsy-based tri-modality sampling using an ultrathin bronchoscope for the diagnosis of peripheral lung lesions: A prospective observational study. Respiration.

[23] Husta B.C., Cheng G.Z., Batra H., Reisenauer J.S., Bartek W.M., Kalchiem-Dekel O., Zouk A., Patel N., Chawla M., Eapen G.A. (2026). Shape-sensing robotic-assisted bronchoscopy with integrated mobile cone-beam CT for small nodules: Results from the prospective multicentre CONFIRM study. Thorax.

[24] Thiboutot J., Kapp C.M., Illei P., Shofer S., Gilbert C.R., DiBardino D., DeMaio A., Sethi S., Wahidi M.M., Benn B.S. (2026). Cryobiopsy vs forceps for bronchoscopic lung biopsy: The FROSTBITE-2 randomized clinical trial. JAMA.

