# Supplementary material for: Diagnostic Yield and Safety of Radial Probe Endobronchial Ultrasound-Guided Transbronchial Lung Cryobiopsy with a Guide Sheath in Pulmonary Lesions < 3 cm
Source: Diagnostics (Basel). 2026 Jun 19;16(12):1912. doi: 10.3390/diagnostics16121912 (PMC13298941; doi:10.3390/diagnostics16121912)
Supplement: Supplementary file 1 [file diagnostics-16-01912-s001.zip › diagnostics-4339139-supplementary.pdf]

Supplementary Table S1. Comparison of Baseline Characteristics Between Diagnosed and Non-Diagnosed Cases

Variable	Diagnostic [a] (n=79)	Non-diagnostic [b] (n=20)	P-value
Age	68.2 (±10.6)	67.7 (±7.8)	0.847
Male sex	40 (50.6%)	12 (60.0%)	0.454
Lobar location			0.307
Upper lung	54 (68.4%)	16 (80.0%)	
Lower lung	25 (31.6%)	4 (20.0%)	
Axial distribution [c]			0.546
Inner	1 (1.3%)	1 (5.0%)	
Middle	44 (55.7%)	10 (50.0%)	
Outer	34 (43.0%)	9 (45.0%)	
Lesion size on CT, cm	2.9 (±1.9)	2.04 (1.1)	0.080
CT-BS	70 (88.6%)	11 (55.0%)	<0.001
Type of lesion, n (%)			
Solid	63 (79.7%)	12 (60.0%)	0.066
Sub-solid	16 (20.3%)	8 (40.0%)	
RP-EBU			
Within	41 (51.9%)	8 (40.0%)	0.530
Eccentric	38 (48.1%)	12 (60.0%)	
Dense sign	55 (69.6%)	9 (45.0%)	0.088
Blizzard sign	24 (30.4%)	11 (55.0%)	

Data are expressed as the mean (±standard deviation) or n (%).

CT-BS; computed tomography bronchus signs, RP-EBUS; radial probe-endobronchial ultrasound

[a] True positive or true negative

[b] False positive, false negative, or indeterminate

[c] Axial distribution was defined as the inner, middle, and outer third
